# Rapid neuroplasticity changes and response to intravenous ketamine: a randomized controlled trial in treatment-resistant depression

**DOI:** 10.1038/s41398-023-02451-0

**Published:** 2023-05-09

**Authors:** Jared Kopelman, Timothy A. Keller, Benjamin Panny, Angela Griffo, Michelle Degutis, Crystal Spotts, Nicolas Cruz, Elizabeth Bell, Kevin Do-Nguyen, Meredith L. Wallace, Sanjay J. Mathew, Robert H. Howland, Rebecca B. Price

**Affiliations:** 1 University of California San Diego School of Medicine, San Diego, CA USA; 2 Carnegie Mellon University, Pittsburgh, PA USA; 3 University of Pittsburgh School of Medicine, Pittsburgh, PA USA; 4 Baylor College of Medicine and Michael E. DeBakey VA Medical Center, Houston, TX USA

**Keywords:** Depression, Hippocampus

## Abstract

Intravenous ketamine is posited to rapidly reverse depression by rapidly enhancing neuroplasticity. In human patients, we quantified gray matter microstructural changes on a rapid (24-h) timescale within key regions where neuroplasticity enhancements post-ketamine have been implicated in animal models. In this study, 98 unipolar depressed adults who failed at least one antidepressant medication were randomized 2:1 to a single infusion of intravenous ketamine (0.5 mg/kg) or vehicle (saline) and completed diffusion tensor imaging (DTI) assessments at pre-infusion baseline and 24-h post-infusion. DTI mean diffusivity (DTI-MD), a putative marker of microstructural neuroplasticity in gray matter, was calculated for 7 regions of interest (left and right BA10, amygdala, and hippocampus; and ventral Anterior Cingulate Cortex) and compared to clinical response measured with the Montgomery-Asberg Depression Rating Scale (MADRS) and the Quick Inventory of Depressive Symptoms-Self-Report (QIDS-SR). Individual differences in DTI-MD change (greater decrease from baseline to 24-h post-infusion, indicative of more neuroplasticity enhancement) were associated with larger improvements in depression scores across several regions. In the left BA10 and left amygdala, these relationships were driven primarily by the ketamine group (group * DTI-MD interaction effects: *p* = 0.016–0.082). In the right BA10, these associations generalized to both infusion arms (*p* = 0.007). In the left and right hippocampus, on the MADRS only, interaction effects were observed in the opposite direction, such that DTI-MD change was inversely associated with depression change in the ketamine arm specifically (group * DTI-MD interaction effects: *p* = 0.032–0.06). The acute effects of ketamine on depression may be mediated, in part, by acute changes in neuroplasticity quantifiable with DTI.

## Introduction

Major depressive disorder is the leading cause of disability adjusted life years (DALYs) in the USA and represents an enormous public health burden [1]. Deficits in neuroplasticity and corresponding deficits in the ability to respond adaptively to the environment are seen in both depressed human subjects and in rodent models of depression-like behavior, and therefore may be a core mechanism underlying the disorder [2]. A diversity of structural and functional deficits in neuroplasticity underlie depression-like behavioral states seen in rodents following stress exposure (e.g., anhedonia-like behavior, tested via the sucrose preference test; anxiety-like behavior, indexed by the novelty-suppressed feeding test; and despair-like behavior, observed during the forced swim test) [3]. These neuroplasticity deficits include decreased long-term potentiation and/or increased long-term depression, decreased synaptic protein expression, impaired BDNF and mTOR signaling, decreased synaptogenesis/atrophy of existing synapses, and decreased neurogenesis/atrophy of neurons, ultimately resulting in dysfunction of corticolimbic circuits and expression of maladaptive behavioral strategies [2, 4–8]. Effective antidepressant therapies have been shown to reverse many of these deficits, further emphasizing the relevance of these studies to depression and providing some of the strongest evidence to date that impairments in neuroplasticity may be a core mechanism underlying depression [9–12].

While functional neuroplasticity cannot be directly tested in the living human brain, post-mortem studies of depression show reductions in markers of neuroplasticity, including reduced BDNF and decreased synapses and synapse-related gene expression [13, 14]. Neuroimaging studies in depressed subjects reveal hypofunction and gray matter volume loss in key corticolimbic structures including the PFC and hippocampus, as well as decreased functional integration across these regions and their associated networks. These neural network alterations are hypothesized to result in deficits in flexible cognition and affective processing that manifest in depressed patients as rigid, maladaptive behavioral responses [15, 16].

Recent evidence has shown that ketamine, a dissociative anesthetic, has rapid and robust antidepressant effects, including in treatment-resistant patients for whom other therapies have been ineffective [17–22]. These results have been widely replicated, but the exact mechanisms by which ketamine reduces depressive symptomology remain unknown. Results in rodent models indicate that a single dose of ketamine induces robust markers of neuroplasticity in depression-relevant brain regions, including increased BDNF release and the stimulation of mTOR signaling in the PFC [23, 24]. In addition, ketamine induces an increase in synapse number and function in the PFC, reversing the loss of specific synapses by stress, an effect that seems necessary for the persistence of its antidepressant-like behavioral effects [12, 24]. In humans, ketamine also alters neural response to emotional stimuli [25] and EEG gamma power [26–29], a putative marker of activity-dependent plasticity.

To our knowledge, no one has specifically linked microstructural neuroplasticity induced by ketamine—akin to the synaptogenesis in the PFC observed in animal models—to its antidepressant effects in humans. While real-time neuroplasticity cannot be directly assessed in the intact human brain, recent advances in neuroimaging have provided evidence that structural remodeling of the human brain can be measured within hours of its occurrence. Several studies have used diffusion tensor imaging (DTI), an MRI-based framework, as an indirect marker of neuroplasticity [30–33]. Mean diffusivity (MD) within gray matter regions, an index derived from DTI, is a measure of water diffusion and therefore tissue density. The generation of new synapses, an important common final pathway of neuroplasticity, results in a decrease in MD, as these novel synapses restrict the flow of water within gray matter [30]. Studies have shown decreases in MD within memory-related brain regions within 2 h of training tasks [30, 31, 33]. This finding was replicated in rodents, and the decrease in DTI-MD was shown to correlate with traditional markers of cellular and molecular neuroplasticity, including increased number of synaptic vesicles, astrocyte activation, and increased expression of synaptic-related proteins, including BDNF [30]. Collectively, these findings in animals and humans specifically tie acute DTI-MD changes to neuroplasticity processes including those involved in synaptogenesis—a prominent mechanism implicated in ketamine’s mechanism of antidepressant action [12, 24].

In the current study, we measured DTI-MD in depression-relevant brain regions (BA10, amygdala, hippocampus, and ventral ACC) before and 24-h following the administration of IV ketamine (0.5 mg/kg) or saline vehicle in depressed human subjects, and related individual differences in these acute neuroplasticity markers to the degree of clinical response 24-h post-infusion. We hypothesized that individual differences in region-specific changes in DTI-MD would predict the response to treatment primarily in patients receiving ketamine, with larger clinical improvements following ketamine tracking with greater degrees of structural change, putatively reflecting neuroplasticity enhancement.

## Methods

This study included secondary analyses of data generated from clinical trial NCT03237286. Study design and primary clinical outcomes have been detailed previously [34]. In the full trial, 154 adult subjects (age 18–60) with moderate to severe depression [Montgomery-Asberg Depression Rating Scale (MADRS [35]) score ≥25] and at least one adequate, failed trial of an FDA-approved antidepressant medication in the current depressive episode (assessed via Antidepressant Treatment Response Questionnaire; ATRQ [36]), were enrolled and randomized to receive either ketamine or vehicle in a 2:1 ratio. An experienced, Master’s-level clinical rater (CRS) administered the MADRS (the primary clinician-rated outcome used for the clinical trial) prior to, and 24 h post-infusion to assess overall depression severity and change following intervention. Similarly, patients self-reported depressive symptoms before and 24-h following ketamine using the Quick Inventory of Depressive Symptoms (QIDS-SR) [37, 38], the primary self-report outcome. A subset of these subjects (31 vehicle, 67 ketamine) had usable DTI neuroimaging data collected prior to and 24-h post-infusion and are included in the current analyses. Subjects received either ketamine (0.5 mg/kg) or vehicle (50 ml 0.9% NaCl) infused over 40-min, as done previously [39–41]^1^. All infusions were administered by blinded, licensed nurses in a medical hospital setting with linked ACLS-certified team, blinded study physician (RHH) oversight, and safety/adverse event monitoring sustained for 4-h post-infusion. Current medications and doses were obtained from subjects via the community treatment form or patient interview with study staff. When there was a discrepancy between these two sources or missing information that could not be resolved via subject follow-up, collateral was obtained from the patient’s electronic medical record. Medication burden was calculated based on the Anti-Depressant Treatment History Form (ATHF) [42–44]. The study was performed at the University of Pittsburgh and approved by the Internal Review Board of the University of Pittsburgh. All participants provided informed consent prior to any study procedure. See Table 1 for descriptive patient characteristics.

**Table 1:** Clinical and demographic characteristics of the sample with usable DTI neuroimaging data.

	Full sample (*n* = 98)		Ketamine (*n* = 67)		Saline (*n* = 31)	
Race/ethnicity						
Non-Hispanic Caucasian, *n* (%)	72	73.5%	47	70.15%	25	80.65%
Hispanic/Latino, *n* (%)	6	6.2%	6	8.96%	0	0.00%
African American, *n* (%)	4	4.1%	2	2.99%	2	6.45%
Asian, *n* (%)	8	8.2%	6	8.96%	2	6.45%
More than one race, *n* (%)	7	7.10%	5	7.46%	2	6.45%
Unknown/incomplete info, *n* (%)	1	1.06	1	1.49%	0	0.00%
Sex						
Assigned female sex at birth, *n* (%)	61	62.2%	40	59.70%	21	67.74%
Taking psychotropic medication, *n* (%)	78	80%	56	83.58%	22	70.97%
Pre-infusion baseline QIDS total score (SD)	15.36	3.99	15.34	4.22	15.39	3.50
Pre-infusion baseline MADRS total score	32.81	5.39	32.84	5.52	32.74	5.20

No variables in the table above differed as a function of treatment group according to unpaired *t*-tests (for continuous variables) or Chi-squared tests (for categorical variables) (*p* > 0.15).

### Neuroimaging acquisition

All neuroimaging data were acquired using a 3T Siemens Prisma and a Siemens 64-channel head coil at the University of Pittsburgh. Diffusion-weighted structural images were acquired using the multi-band sequences (version R016A) provided by the University of Minnesota Center for Magnetic Resonance Research (https://www.cmrr.umn.edu/multi band/). Diffusion-weighted images were collected as oblique-axial scans aligned with the anterior commissure–posterior commissure (AC–PC) line at midline with the monopolar cmrr_mbep2d_diff sequence (http://www.cmrr.umn.edu/multi band) in 72 slices (an ascending interleaved acquisition with 2.0-mm-thick slices and no inter-slice gap). The matrix was 104 × 104 and FOV was 208 mm, resulting in 2.0-mm isotropic voxels (TR = 2443 ms, TE = 88.0 ms, multi-band acceleration factor = 4, number of diffusion encoded directions = 30, diffusion *b* value = 1000 s/mm^2^, number of non-diffusion-encoded images = 4, bandwidth = 2004 Hz/pixel, partial Fourier factor of 7/8). The 30 diffusion encoding vectors were taken from a standard Siemens gradient table. Two sets of these images with opposite phase encoding directions (anterior -> posterior and posterior -> anterior) were collected for each participant in each scanning session (baseline and 24-h). A high-resolution structural scan was also acquired at the baseline scan session (axial MPRAGE: TR = 2400; TE = 2.22; 208 slices; flip angle = 8°; 0.8 mm isotropic voxels).

### Neuroimaging processing

Images were preprocessed in FSL v. 5.0 (https://fsl.fmrib.ox.ac.uk/fsl/fslwiki) following a standard DTI pipeline [33] that included the following steps: correction of geometric distortions (topup), motion (mcflirt), and eddy currents (eddy); linear co-registration of baseline and 24-h data (flirt); non-linear warping to MNI space (fsl_reg to “FMRIB58_FA_1mm” template); and fitting a weighted-least squared diffusion tensor model (dtifit) to produce voxel-wise maps of mean diffusivity at each scan session. A DTI-MD change score (∆-MD = MD pre-infusion – MD at 24 h) was then calculated for each of the following ROIs: left and right BA10, left and right amygdala, left and right hippocampus, and ventral anterior cingulate cortex (vACC; encompassing subgenual and perigenual ACC). These specific regions were selected for analysis based on a priori hypotheses about the role of these regions in both the antidepressant response to ketamine (which has particularly implicated hippocampus and medial PFC areas, including bilateral BA10 and vACC; see [2]) and in depression pathophysiology at large, which additionally establishes a prominent role for the amygdala in depressed patients’ altered patterns of affective processing (reviewed in [2]). Anatomical masks for each ROI were constructed using the MNI atlas and applied to extract the MD score for each participant as an average of all voxels within each ROI. A higher ∆-MD score corresponds to lower mean diffusivity following the infusion, and putatively greater plasticity/synaptogenesis [30]. A Winsorizing procedure was used to rescale extreme ∆-MD values prior to analysis, as described previously [45].

All statistics were run using IBM SPSS Statistics (version 28.0.1.0). To test for the effect of group (ketamine vs. vehicle) on ∆ depression scores and ∆-MD, two-tailed independent sample *t*-tests were performed. Multiple linear regression was performed to predict the improvement in depression (measured as change in MADRS and QIDS-SR scores) from ∆-MD and group (ketamine vs. vehicle), as well as their interaction—enabling formal assessment of any distinct relationships between neuroplasticity and clinical improvement that were evident in the ketamine vs. vehicle arms. Parallel, exploratory analyses of three additional outcome measures (measuring anxiety, positive affect, and dissociative side effects) are presented in the online Supplement.

## Results

### Main effects of treatment group

In the full clinical trial sample, ketamine significantly improved MADRS (*t*(150) = −4.55, *p* < 0.001) and QIDS (*t*(148) = −2.60, *p* = 0.010) scores 24-h post-infusion. In the subset of patients with DTI neuroimaging data, ketamine significantly improved MADRS scores 24-h post-infusion (*t*(96) = −3.01, *p* = 0.003), while there was no significant effect of ketamine on change in QIDS-SR score 24-h post-infusion in this subset of patients (*t*(95) = −1.10, *p* = 0.28). Ketamine had no significant main effect on either raw or Winsorized ∆-MD values in any of the regions investigated.

### Moderation of clinical outcomes by rapid change in MD

Linear regression was performed to predict the improvement in QIDS and MADRS from ∆-MD values, group (ketamine vs. vehicle), and their interaction. Results for all regions are shown in Table 2.

**Table 2:** Interaction term (group * change in mean diffusivity) statistics and robustness of findings when utilizing clinician-administered (MADRS) vs. self-report (QIDS) outcomes and when including covariates in models.

Region	No covariate	Covarying TRD severity	Covarying medication burden	Covarying pre-MD
MADRS				
L BA10	*∆R*^2^ = 0.029; *β* = 0.308; *t*_96_ = 1.760; *p* = 0.082	*∆R*^2^ = 0.022; *β* = 0.267; *t*_96_ = 1.534; *p* = 0.128	*∆R*^2^ = 0.028; *β* = 0.302; *t*_96_ = 1.716; *p* = 0.089	*∆R*^2^ = 0.035; *β* = 0.290; *t*_96_ = 1.641; *p* = 0.104
R BA10	*∆R*^2^ = 0.000; *β* = 0.019; *t*_96_ = 0.109; *p* = 0.913	*∆R*^2^ = 0.002; *β* = −0.083; *t*_96_ = −0.474; *p* = 0.637	*∆R*^2^ = 0.000; *β* = 0.013; *t*_96_ = 0.078; *p* = 0.938	*∆R*^2^ = 0.001; *β* = −0.043; *t*_96_ = −0.250; *p* = 0.803
L Amygdala	*∆R*^2^ = 0.045; *β* = 0.414; *t*_96_ = 2.238; *p* = 0.028	*∆R*^2^ = 0.055; *β* = 0.459; *t*_96_ = 2.518; *p* = 0.014	*∆R*^2^ = 0.043; *β* = 0.410; *t*_96_ = 2.188; *p* = 0.031	*∆R*^2^ = 0.044; *β* = 0.414; *t*_96_ = 2.13; *p* = 0.029
R Amygdala	*∆R*^2^ = 0.001; *β* = −0.046; *t*_96_ = −0.255; *p* = 0.799	*∆R*^2^ = 0.000; *β* = −0.028; *t*_96_ = −0.158; *p* = 0.874	*∆R*^2^ = 0.001; *β* = −0.049; *t*_96_ = −0.270; *p* = 0.788	*∆R*^2^ = 0.001; *β* = −0.048; *t*_96_ = −0.266; *p* = 0.791
L Hippocampus	*∆R*^2^ = 0.044; *β* = −0.337; *t*_96_ = −2.178; *p* = 0.032	*∆R*^2^ = 0.048; *β* = −0.351; *t*_96_ = −2.315; *p* = 0.023	*∆R*^2^ = 0.044; *β* = −0.338; *t*_96_ = −2.175; *p* = 0.032	*∆R*^2^ = 0.045; *β* = −0.343; *t*_96_ = −2.202; *p* = 0.030
R Hippocampus	*∆R*^2^ = 0.033; *β* = −0.303; *t*_96_ = −1.902; *p* = 0.060	*∆R*^2^ = 0.035; *β* = −0.310; *t*_96_ = −1.986; *p* = 0.050	*∆R*^2^ = 0.034; *β* = −0.308; *t*_96_ = −1.923; *p* = 0.058	*∆R*^2^ = 0.033; *β* = −0.304; *t*_96_ = −1.897; *p* = 0.061
vACC	*∆R*^2^ = 0.001; *β* = −0.043; *t*_96_ = −0.254; *p* = 0.800	*∆R*^2^ = 0.000; *β* = −0.032; *t*_96_ = −0.193; *p* = 0.847	*∆R*^2^ = 0.001; *β* = −0.042; *t*_96_ = −0.246; *p* = 0.806	*∆R*^2^ = 0.001; *β* = −0.055; *t*_96_ = −0.322; *p* = 0.748
QIDS				
L BA10	*∆R*^2^ = 0.045; *β* = 0.386; *t*_96_ = 2.143; *p* = 0.035	*∆R*^2^ = 0.037; *β* = 0.352; *t*_96_ = 1.952; *p* = 0.054	*∆R*^2^ = 0.043; *β* = 0.377; *t*_96_ = 2.084; *p* = 0.040	*∆R*^2^ = 0.046; *β* = 0.382; *t*_96_ = 2.094; *p* = 0.039
R BA10	*∆R*^2^ = 0.016; *β* = 0.238; *t*_96_ = 1.305; *p* = 0.195	*∆R*^2^ = 0.007; *β* = 0.160; *t*_96_ = 0.855; *p* = 0.395	*∆R*^2^ = 0.016; *β* = 0.233; *t*_96_ = 1.276; *p* = 0.205	*∆R*^2^ = 0.010; *β* = 0.212; *t*_96_ = 1.144; *p* = 0.256
L Amygdala	*∆R*^2^ = 0.059; *β* = 0.477; *t*_96_ = 2.446; *p* = 0.016	*∆R*^2^ = 0.070; *β* = 0.521; *t*_96_ = 2.711; *p* = 0.008	*∆R*^2^ = 0.056; *β* = 0.465; *t*_96_ = 2.359; *p* = 0.020	*∆R*^2^ = 0.050; *β* = 0.440; *t*_96_ = 2.261; *p* = 0.026
R Amygdala	*∆R*^2^ = 0.001; *β* = −0.063; *t*_96_ = −0.333; *p* = 0.740	*∆R*^2^ = 0.001; *β* = −0.047 *t*_96_ = −0.254; *p* = 0.800	*∆R*^2^ = 0.001; *β* = −0.068; *t*_96_ = −0.360; *p* = 0.719	*∆R*^2^ = 0.002; *β* = −0.072; *t*_96_ = −0.392; *p* = 0.696
L Hippocampus	*∆R*^2^ = 0.001; *β* = −0.061; *t*_96_ = −0.353; *p* = 0.725	*∆R*^2^ = 0.002; *β* = −0.065; *t*_96_ = −0.383; *p* = 0.702	*∆R*^2^ = 0.001; *β* = −0.060; *t*_96_ = −0.351; *p* = 0.727	*∆R*^2^ = 0.002; *β* = −0.070; *t*_96_ = −0.410; *p* = 0.683
R Hippocampus	*∆R*^2^ = 0.000; *β* = −0.011; *t*_96_ = −0.063; *p* = 0.950	*∆R*^2^ = 0.000; *β* = −0.010; *t*_96_ = −0.059; *p* = 0.953	*∆R*^2^ = 0.000; *β* = −0.017; *t*_96_ = −0.096; *p* = 0.924	*∆R*^2^ = 0.000; *β* = −0.007; *t*_96_ = 0.042; *p* = 0.967
vACC	*∆R*^2^ = 0.005; *β* = 0.124; *t*_96_ = 0.695; *p* = 0.489	*∆R*^2^ = 0.006; *β* = 0.140; *t*_96_ = 0.793; *p* = 0.430	*∆R*^2^ = 0.005; *β* = 0.128; *t*_96_ = 0.713; *p* = 0.478	*∆R*^2^ = 0.005; *β* = 0.118; *t*_96_ = 0.654; *p* = 0.515

The values provided for *∆R*^2^*, β*, *t*, and *p* in each column above all uniformly correspond to the addition of the group * *∆*MD interaction term (for the specified region) into regression models which also contain main effects of group and *∆*MD in predicting % improvement in the MADRS (top half of table) and QIDS (bottom half of table). The “No Covariate” column reflects the statistics for primary models, as reported in “Results” section text; the additional 3 columns to the right reflect the impact on the interaction term statistics when also including each specified variable as a covariate in models.

#### L BA10

**Fig. 1:**
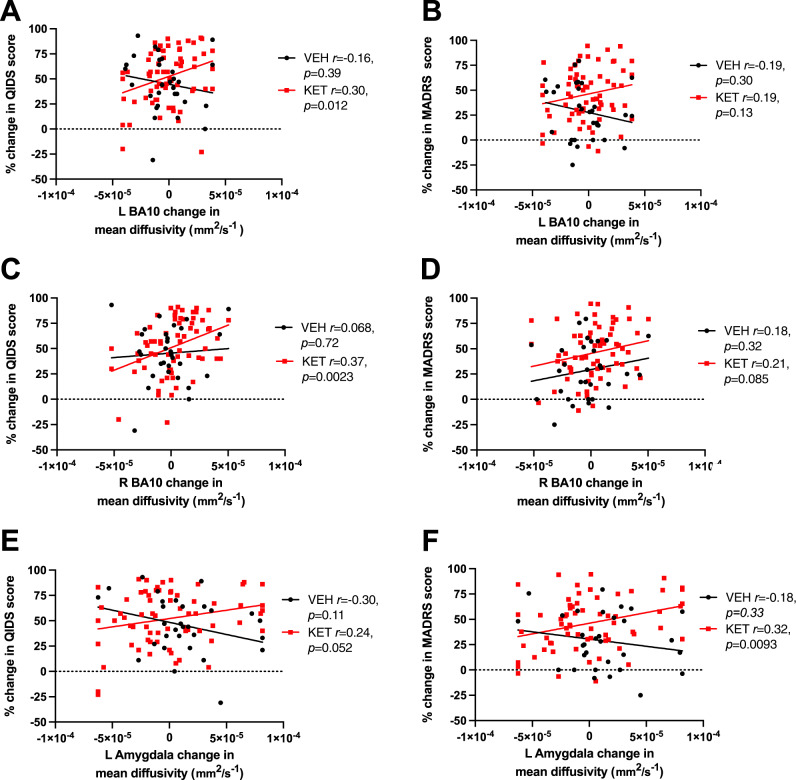
Associations between change in depression and change in mean diffusivity as a function of treatment. Within-group correlations between change in QIDS-SR (**A**, **C**, **E**) or MADRS (**B**, **D**, **F**) scores and the change in mean diffusivity for select brain regions. Post hoc Pearson *r* and *p* values shown separately for the vehicle (*n* = 31) and ketamine (*n* = 67) groups; see main text and Table 2 for full omnibus regression statistics. A positive change in depression score indicates an improvement in depression on that scale, and a positive change in MD indicates decreased diffusion in that region (interpreted as increased plasticity).

For QIDS scores, there was a significant ∆-MD * group interaction effect, such that decreased MD scores (i.e., putative neuroplasticity increase) predicted greater improvement in QIDS scores in the ketamine group specifically (*β* = 0.386, *t*(96) = 2.143, *p* = 0.035; Fig. 1A). For MADRS scores, there was a significant main effect of group (*β* = 0.314, *t*(96) = 3.207, *p* = 0.002) and a trend for a ∆-MD * group interaction effect (*β* = 0.308, *t*(96) = 1.760, *p* = 0.082; Fig. 1B). Greater improvement in MADRS scores was associated with a reduction in MD, and this relationship was particularly evident in the ketamine group.

#### R BA10

There was a significant main effect of ∆-MD on QIDS (∆-MD: *β* = 0.275, *t*(96) = 2.771, *p* = 0.007; Fig. 1C) and a significant main effect of both ∆-MD and group on MADRS scores (∆-MD: *β* = 0.196, *t*(96) = 2.019, *p* = 0.046; group: *β* = 0.268, *t*(96) = 2.763 *p* = 0.007; Fig. 1D), such that reduced MD predicted greater improvement in depression score (independent of group). There was no significant ∆-MD * group interaction effect in this region for either QIDS or MADRS (*p* ≥ 0.195).

#### L amygdala

There was a significant ∆-MD * group interaction effect on both QIDS (*β* = 0.477, *t*(96) = 2.446, *p* = 0.016; Fig. 1E) and MADRS (*β* = 0.414, *t*(96) = 2.238, *p* = 0.028; Fig. 1F) such that decreases in MD predicted greater improvement in depression in the ketamine group specifically.

#### L hippocampus

**Fig. 2:**
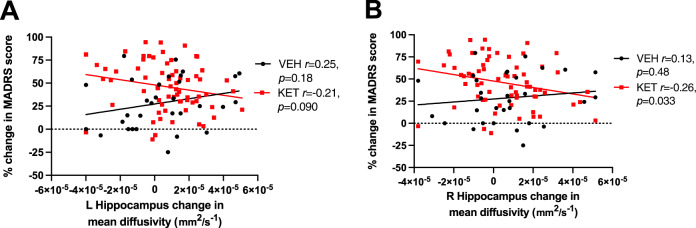
Associations between change in depression and change in hippocampal mean diffusivity as a function of treatment. Within-group correlations between change in MADRS score and the change in mean diffusivity for left (**A**) and right (**B**) hippocampus. Post hoc Pearson *r* and *p* values shown separately for the vehicle (*n* = 31) and ketamine (*n* = 67) groups; see main text and Table 2 for full omnibus regression statistics. A positive change in depression score indicates an improvement in depression on the MADRS, and a positive change in MD indicates decreased diffusion in that region (interpreted as increased plasticity).

For the MADRS only, there was a significant ∆-MD * group interaction effect (*β* = −0.337, *t*(96) = −2.178, *p* = 0.032; Fig. 2A). The directionality of these results is opposite to those in left BA10 and left amygdala above, such that increased MD predicted improved MADRS score in the ketamine group. There were no significant main or interaction effects predicting improvement in QIDS score (*p* > 0.05).

#### R hippocampus

For the MADRS only, there were significant main effects of group (*β* = 0.343, *t*(96) = 3.420, *p* = 0.001) and a trend-level ∆-MD * group interaction in right hippocampus (*β* = −0.303, *t*(96) = −1.902, *p* = 0.060; Fig. 2B). Like in the left hippocampus, increased MD in the right hippocampus predicted improved MADRS score following ketamine. There were no significant main or interaction effects predicting improvement in QIDS score (*p* > 0.05).

#### Other regions examined and sensitivity analyses

There were no significant main or interaction effects in the right amygdala or the vACC.

Sensitivity analyses were performed to assess robustness of interaction effect findings when including the following covariates: baseline DTI-MD score, severity of treatment resistance [moderate (<3 failed adequate trials) vs. severe], and use of concurrent psychotropic medications (see Table 2). Inclusion of each covariate into our statistical models had minimal impact on the pattern and statistical significance of the findings described above.

## Discussion

In the present study, we found an association between change in DTI-MD (∆-MD), a putative marker of neuroplasticity, and treatment response to ketamine in a sample of patients with depression. Reductions in MD scores in left BA10 and left amygdala, representing putative increased plasticity in these regions, predicted greater improvement in depression scores specifically in patients receiving ketamine. In right BA10, decreased MD scores predicted greater improvement in depression scores, independent of group (as reflected in the lack of a significant group * ∆-MD interaction effect). In both right and left hippocampus, the results were paradoxically in the opposite direction, with higher ∆-MD scores predicting greater improvement in clinician-rated depression scores in subjects receiving ketamine. However, there was no similar interaction between ∆-MD and group in predicting self-reported depression scores in either left or right hippocampus. Lastly, we found no significant effect of ∆-MD on depression scores in vACC or right amygdala. These findings were not appreciably changed in sensitivity analyses that included covariates in the statistical models, including concomitant psychiatric medication burden, level of treatment resistance, and baseline DTI-MD values (Table 2).

To our knowledge, this is the first placebo-controlled human study to investigate the association between a structural marker of acute neuroplasticity in depression-relevant brain regions and treatment response to ketamine, providing a first attempt to translate animal neuroscience findings, which have widely implicated synaptogenic mechanisms of action, back to the clinic. The regions in which increased plasticity predicted change in depression scores are right and left BA10 and left amygdala. Activity in these regions has been shown to be altered in depressed subjects, with studies of BA10 (and nearby related PFC regions) showing hyperactivation to reward-related cues and hypoactivation to negative affective cues (e.g., negatively valanced faces) in depressed subjects relative to controls [46]. Furthermore, a large body of literature in depressed subjects shows hyperactivation of the amygdala in response to negative affective cues and hypoactivation in response to positive affective cues (e.g., positively valanced faces) relative to controls [47]. Antidepressants may also change activity in these regions, with a meta-analysis showing that the neural biases in emotional processing in depressed subjects in the PFC and amygdala were normalized following the administration of an SSRI [48]. Some more recent evidence indicates that this may be true for treatment with ketamine as well, with decreased amygdala activity in response to emotionally salient stimuli being perhaps the most well-replicated finding in the ketamine neuroimaging literature [49–52]. Many previous neuroimaging studies of response to ketamine have also shown changes in functional connectivity across brain networks, and while this literature is expansive and varied, studies have consistently implicated functional connectivity involving BA10 and related PFC regions and the amygdala in the antidepressant response to ketamine [51, 53–56]. Interestingly, the laterality of regions involved in ketamine response in human neuroimaging research varies from study-to-study, with the exception of the left amygdala, which, as in our current study, is consistently implicated more commonly than the right amygdala [50].

The structural mechanisms that underlie the functional changes in BA10 and left amygdala described above are unknown, but likely involve remodeling at the circuit, cellular, and/or synaptic level. This type of remodeling alters brain microstructure, leading to a change in the diffusion properties of water within this region (i.e., diffusion is restricted), resulting in a decrease in MD that reflects increased neuroplasticity [30–32]. The changes we observed in BA10 and left amygdala following ketamine administration could represent a structural correlate of the antidepressant behavioral effect of the drug. Ketamine rapidly alters neural and behavioral responses to incoming stimuli, with changes in how brain regions respond to emotional stimuli and accompanying changes in behavioral output [29, 49, 50, 52]. One way by which this could happen is the rearrangement of multi-synaptic connections between sensory input and behavioral output, reflected in this study as a decrease in MD. The amygdala in particular plays an important role in responding to emotional stimuli and regulating behavioral output. Structural plasticity in this region might thus be readily leveraged to reduce the salience of negative stimuli and increase the salience of positive emotional stimuli. Through further testing of this hypothesis, the molecular effects of ketamine might be traced to its neural and behavioral effects in human patients.

While in left BA10 there was a significant ∆-MD × group interaction effect, there was no similar interaction effect in the right BA10. This could indicate that plasticity in this region may play a larger role in the non-specific antidepressant effects that are common between the ketamine and placebo control groups, such as those associated with the infusion/research protocol, including the formation of therapeutic relationships, being in a therapeutic environment, positive feelings of helping others by contributing to research, and expectation of improvement.

In contrast to the results seen in BA10 and left amygdala, which conformed to expectations based on the animal literature, in both the left and right hippocampus there was a significant effect in the opposite direction, with increased MD being associated with improved clinician-rated depression scores in the ketamine group. Unlike the majority of other findings we report, which were largely robust across both clinician- and self-rated depression symptoms (e.g., Fig. 1 and Table 2), this pattern did not generalize to the QIDS, and may therefore be less reliable. While a decrease in MD is assumed to represent increased plasticity, with some mechanistic data to support this [30], the interpretation of increased MD is less straightforward. Increased MD could represent an inverse mechanism of the decrease in MD, i.e., increased MD = decreased plasticity. If this is the case, it may be that ketamine causes selective pruning of hippocampal synapses (e.g., those associated with maladaptive behavioral responses). However, it seems unlikely that an overall decrease in plasticity of the hippocampus would be associated with improved depression scores following ketamine, especially in a sample of depressed subjects where low levels of hippocampal plasticity and structural integrity are posited to occur at baseline. Other interpretations are potentially more likely and are not mutually exclusive. Increased MD has been associated with specific behavioral states, including higher empathizing and cooperativeness [57], which might be more likely to occur in patients with more improved depression scores. It has also previously been reported that MD increases in response to increases in cerebral blood flow (CBF) [58–60]. There is some evidence that improvement in particular symptom domains, namely anhedonia, is positively correlated with regional glucose metabolic rates, and we cannot rule out CBF increases being similarly associated with antidepressant response as well as increased MD. Other causes of increased MD, such as changes in dopamine or iron content of tissue, atrophy, or inflammation are possible, but less likely to be occurring in the hippocampus 24-h following the administration of ketamine [30, 57, 58, 61].

Finally, we did not observe any relationships between clinical outcomes and ∆-MD in two other regions included in analyses: right amygdala and vACC. Relative to the other regions under examination, the specific neural processes captured via DTI-MD within these two regions may play a less foundational role in ketamine’s rapid antidepressant effects; although we cannot rule out other possible explanations, such as measurement error, insufficient sample size, and other contributors to Type II error.

Our data have clinical implications for the use of ketamine for depression. The plasticity induced by ketamine seems to persist, at least out to 24-h post-infusion, and potentially well beyond this. We can take advantage of this period of increased plasticity to augment the antidepressant effects of ketamine by combining it with other therapies, such as cognitive training. Indeed, in the primary outcome for this clinical trial, we previously reported that Automated Self-Association Training—a novel, low-cost, brief, computer-based intervention—extended the rapid antidepressant effect of a single ketamine infusion for at least 30 days [34]. In addition to this potential for combinatorial, synergistic treatments, this study also points to the potential importance of the environment following treatment. If the brain is in a plastic state following ketamine, then the potential impact of the environment over the next 24+ h could be outsized, with a therapeutic environment potentially strengthening the effects of ketamine and a stressful environment potentially weakening them.

There are some limitations to the current study. Our measure of acute neuroplasticity, ∆-MD, is by necessity an indirect measure. While the previous studies using this metric as a measure of acute plasticity were investigating changes in hippocampal MD following learning, we are using it as a proxy for neuroplasticity in the context of treatment response for psychiatric disorders. Other factors that affect the diffusion of water, such as inflammation, could serve as confounds for our interpretation. The study was also limited by sample size, with 31 vehicle and 67 ketamine subjects, which is a subset of the whole clinical trial, and was likely underpowered to detect some interaction effects suggested by visual plots (Fig. 1), as well as a main effect of ketamine on QIDS scores, an effect that was present in the whole sample. The difference in sample sizes also makes us better powered to find significant post hoc correlations between changes in depression score and ∆-MD in specific regions in the ketamine group relative to the saline group. In an effort to balance between Type I and Type II error risk, we selected a constrained set of 7 regions a priori and did not adjust for multiple comparisons within this confined set. Our study also did not include individuals without depression, precluding the ability to assess for baseline group differences in DTI-MD as well as normalization of any such differences following ketamine. Changes in MD in several regions significantly predicted improvement in depression scores, however the effect sizes were relatively small, with *R*^2^ values between 0.05 and 0.18. Lastly, there was no main effect of ketamine on ∆-MD, as we might predict if it were the sole mediator of treatment response, indicating that numerous other factors are likely involved in the antidepressant effects of ketamine among human patients. Such factors could include additional neuroplasticity mechanisms, such as the impact of ketamine on white matter microstructure and tractography [62, 63] which was not assessed in the current study due to scanner sequence timing constraints and lack of a priori hypotheses regarding white matter effects; as well as a wide range of factors beyond neuroplasticity per se.

## Conclusion

In the past two decades, ketamine has emerged as one of the most promising pharmacological treatments for depression since selective serotonin re-uptake inhibitors (SSRIs) became available. While the exact mechanism is unknown, it has been hypothesized that ketamine’s antidepressant effects are at least in part mediated by increases in neuroplasticity broadly, and by synaptogenic actions specifically. We found that a proxy for structural neuroplasticity was associated with treatment response to ketamine in a subset of the depression-relevant brain regions we examined. These results have important implications for the development of synergistic therapies and for understanding the neurobiological mechanisms by which ketamine exerts rapid antidepressant actions in depressed patients.

## Supplementary information

Online Supplement

## Footnotes

1 *N* = 28 patients were enrolled prior to adding the DTI sequence to the MRI protocol; *N* = 23 had unusable DTI data due to a scanner acquisition error at one or more timepoints; *N* = 3 did not complete DTI sequence due to scanner time constraints; *N* = 2 patients did not return to complete 24-h MRI scan.
