# Supplementary material for: Rapid neuroplasticity changes and response to intravenous ketamine: a randomized controlled trial in treatment-resistant depression
Source: Transl Psychiatry. 2023 May 9;13:159. doi: 10.1038/s41398-023-02451-0 (PMC10170140; doi:10.1038/s41398-023-02451-0)
Supplement: Supplementary file 1 — Online Supplement [file 41398_2023_2451_MOESM1_ESM.docx]

**Online Supplement**

Kopelman et al, “Rapid Neuroplasticity Changes and Response to Intravenous Ketamine: A Randomized Controlled Trial in Treatment-Resistant Depression”

Exploratory outcomes. Secondary self-report measures collected at baseline and 24-hours post-infusion included the PROMIS adaptive test of anxiety (T-score) and the NEUROQOL adaptive test of Positive Affect (T-score). These symptom scores were converted to % change from baseline, as described in the main text. To assess acute dissociative side effects, the Clinician-Administered Dissociative Symptoms Scale (CADSS) administered by a trained rater at 40-minutes post-infusion. All three outcomes were significantly associated with treatment group (ketamine vs. saline: *p*’s <.001 per unpaired t-tests). These three additional outcome measures were then explored in parallel regression analyses, as described in the main text, to uncover any significant relationships between the neuroplasticity marker (change in DTI-MD) and these exploratory outcomes.

These analyses revealed several significant findings for either the main effect of *∆*MD or an interacting effect of group * *∆*MD on these exploratory outcomes. In both the L and R BA 10, a main effect linking greater *∆*MD to greater improvement (increase from baseline) in positive affect across both treatment groups was observed (L BA10: Beta=-.22, *p*=.024; R BA10: Beta=-.23, *p*=.019). In the R BA 10, there was also a main effect linking greater *∆*MD to greater improvement in anxiety across both treatment groups (Beta=.28, *p*=.006).

In the left amygdala, there was a significant group**∆*MD interaction effect predicting improvements in positive affect (interaction term: Beta=-.52, *p*=.007; *∆*R^2^=.07). Similar to the pattern of findings described in the main text, increased MD predicted improved positive affect in the ketamine group, while an opposing relationship was observed in the saline group.

No significant main or interaction effects were observed in the remaining *a priori* regions (right amygdala, R/L hippocampus, vACC), nor were any findings observed relating *∆*MD in any of the 7 regions to CADSS dissociation scores during the infusion.
